# Comparison between MostCare and echocardiography for cardiac output estimation in trauma patients

**DOI:** 10.1186/cc9478

**Published:** 2011-03-11

**Authors:** E Falciani, F Franchi, R Silvestri, L Cubattoli, P Mongelli, E Casadei, P Giomarelli, S Scolletta

**Affiliations:** 1 University of Siena, Italy

## Introduction

The reliability of the pulse contour methods (PCMs) in cardiac output (CO) monitoring has been questioned when changes in arterial tone occur spontaneously (for example, pain, hypovolemia) or after a therapeutic intervention (for example, nitroglycerin, nor-epinephrine). The purpose of this study was to compare the CO values assessed with the MostCare system (Vygon, Padova, Italy) (MC-CO) with those obtained with transthoracic echocardiography (Esaote Mylab 70, Genova, Italy) (TTE-CO) in trauma patients treated with norepinephrine.

## Methods

Twenty-seven adult trauma patients admitted to a seven-bed ICU and requiring norepinephrine infusion were enrolled in the study. Inclusion criteria were: age >18, no aortic valve pathologies, sinus rhythm. TTE-CO and MC-CO were evaluated simultaneously at two different stable hemodynamic states: baseline (T1), and after raising mean arterial pressure to 90 mmHg by starting norepinephrine infusion (T2). The MostCare system, an uncalibrated PCM, was connected directly to the main monitor of the patient for the analysis of the radial artery pressure wave. Bland-Altman and linear regression analyses were performed.

## Results

Fifty-four paired CO values were obtained; TTE-CO values ranged from 2.9 to 6.8 l/minute and MC-CO from 2.8 to 6.9 l/minute. At T1 the mean bias between the techniques was -0.07 l/minute (2SD = 0.69 l/minute), with a percentage of error (PE) of 15% and *R *= 0.9; at T2 the mean bias between the techniques was -0.13 l/minute (2SD = 0.83 l/minute), PE was 17% and *R *= 0.88. Overall, a good correlation between TTE-CO and MC-CO was observed (*R *= 0.9, *P *< 0.01), with a mean bias of -0.10 l/minute (2SD ± 0.76 l/minute), 95% limits of agreement of -0.86 to 0.66 l/minute, and a PE of 16%. Mean arterial pressure was 82.2 ± 11.6 mmHg at T1 and 94.1 ± 3.8 mmHg at T2 (*P *< 0.05). Heart rate did not change significantly from T1 to T2 (78.9 ± 13.6 bpm vs. 78.3 ± 18.7 bpm, respectively, *P *> 0.05). Mean dosage of norepinephrine was 0.22 ± 0.1 μg/kg/minute (range 0.1 to 0.65 μg/kg/minute).

## Conclusions

MC-CO values showed a good agreement with TTE-CO at the two different hemodynamic states of trauma patients. Under the studied conditions, the reliability of the MostCare system seemed not to be affected by the changes in vascular tone induced by norepinephrine infusion.

